# 6,6′-Diamino-1,1′,3,3′-tetra­methyl-5,5′-(4-chloro­benzyl­idene)bis­[pyrimidine-2,4(1*H*,3*H*)-dione]

**DOI:** 10.1107/S1600536809035818

**Published:** 2009-09-09

**Authors:** Subrata Das, Binoy K. Saikia, Babulal Das, Lakhinath Saikia, Ashim J. Thakur

**Affiliations:** a Department of Chemical Sciences, Tezpur University, Tezpur 784 028, India; b Department of Chemistry, Indian Institute of Technology Guwahati, Guwahati 781039, India

## Abstract

The title compound, C_19_H_21_ClN_6_O_4_, is a 1:2 adduct of *p*-chloro­benzaldehyde and uracil. It crystallizes with two mol­ecules in the asymmetric unit. The two uracil units in the same mol­ecule are connected by a pair of strong N—H⋯O hydrogen bonds. The packing is stabilized by N—H⋯O, C—H⋯O and C—H⋯N inter­actions.

## Related literature

For the biological activity and medicinal applications of heterocyclic compounds, especially pyrimidine derivatives, see: Zheng *et al.* (2007 ▶); Jain *et al.* (2006 ▶). The title compound was synthesized from 6-amino- 1,3-dimethyl­pyrimidine-2,4(1*H*, 3H)-dione, which is an important building block, see: Blicke & Godt (1954 ▶); Das *et al.* (2008 ▶).
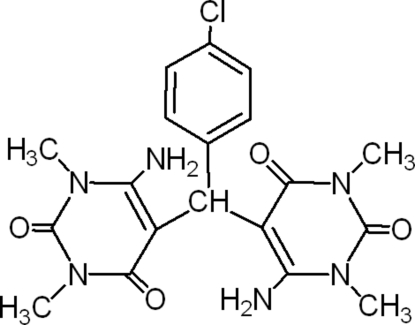


## Experimental

### 

#### Crystal data

C_19_H_21_ClN_6_O_4_
*M* *_r_* = 432.87
Orthorhombic, 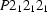

*a* = 11.5365 (3) Å
*b* = 14.5935 (4) Å
*c* = 23.2102 (6) Å
*V* = 3907.62 (18) Å^3^
*Z* = 8
Mo *K*α radiation
μ = 0.24 mm^−1^
*T* = 293 K
0.40 × 0.28 × 0.16 mm

#### Data collection

Bruker SMART CCD area-detector diffractometer
Absorption correction: none
53787 measured reflections
9742 independent reflections
6901 reflections with *I* > 2σ(*I*)
*R*_int_ = 0.038

#### Refinement

*R*[*F* ^2^ > 2σ(*F* ^2^)] = 0.056
*wR*(*F* ^2^) = 0.166
*S* = 1.06
9742 reflections
549 parameters
H-atom parameters constrained
Δρ_max_ = 1.27 e Å^−3^
Δρ_min_ = −0.28 e Å^−3^
Absolute structure: Flack (1983 ▶), 4345 Friedel pairs
Flack parameter: 0.15 (8)

### 

Data collection: *SMART* (Bruker, 2001 ▶); cell refinement: *SAINT* (Bruker, 2001 ▶); data reduction: *SAINT*; program(s) used to solve structure: *SHELXS97* (Sheldrick, 2008 ▶); program(s) used to refine structure: *SHELXL97* (Sheldrick, 2008 ▶); molecular graphics: *XP* in *SHELXTL* (Sheldrick, 2008 ▶); software used to prepare material for publication: *PLATON* (Spek, 2009 ▶).

## Supplementary Material

Crystal structure: contains datablocks I, global. DOI: 10.1107/S1600536809035818/bt5045sup1.cif

Structure factors: contains datablocks I. DOI: 10.1107/S1600536809035818/bt5045Isup2.hkl

Additional supplementary materials:  crystallographic information; 3D view; checkCIF report

## Figures and Tables

**Table 1:** Hydrogen-bond geometry (Å, °)

*D*—H⋯*A*	*D*—H	H⋯*A*	*D*⋯*A*	*D*—H⋯*A*
N3—H3*A*⋯O4	0.86	2.13	2.966 (4)	164
N3—H3*B*⋯O1^i^	0.86	2.26	3.059 (3)	154
N6—H6*A*⋯O6^i^	0.86	2.29	3.041 (3)	146
N6—H6*B*⋯O1	0.86	1.93	2.761 (3)	161
N9—H9*A*⋯O4^ii^	0.86	2.51	3.243 (4)	144
N9—H9*B*⋯O8	0.86	1.98	2.799 (3)	159
N12—H12*A*⋯O6	0.86	2.09	2.924 (3)	163
N12—H12*B*⋯O8^ii^	0.86	2.32	3.106 (3)	153
C1—H1⋯O4	0.98	2.22	2.794 (4)	116
C1—H1⋯N3	0.98	2.41	2.887 (4)	109
C12—H12*C*⋯O1^i^	0.96	2.45	3.164 (4)	131
C12—H12*D*⋯O2	0.96	2.27	2.708 (4)	107
C12—H12*D*⋯O5^iii^	0.96	2.60	3.322 (4)	133
C13—H13*A*⋯O2	0.96	2.36	2.713 (4)	101
C18—H18*B*⋯O4	0.96	2.24	2.673 (6)	106
C19—H19*B*⋯O3	0.96	2.23	2.650 (5)	105
C20—H20⋯O6	0.98	2.23	2.807 (4)	116
C20—H20⋯N12	0.98	2.41	2.876 (4)	108
C31—H31*B*⋯O5	0.96	2.27	2.715 (5)	108
C32—H32*A*⋯O5	0.96	2.33	2.711 (5)	103
C37—H37*A*⋯O8^ii^	0.96	2.57	3.208 (4)	124
C37—H37*C*⋯O7	0.96	2.28	2.707 (4)	106
C38—H38*B*⋯O8	0.96	2.27	2.699 (4)	106

Symmetry codes: (i) 
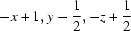
; (ii) 
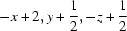
; (iii) 
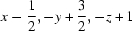
.

**Fig. 1.**
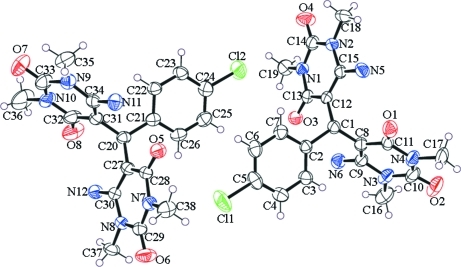
An ORTEP view of the title compound with the atom numbering scheme. Displacement ellipsoids are drawn at the 30% probability level and H atoms are shown as small spheres of arbitrary radii.

## supplementary crystallographic information

### Comment

Heterocyclic compounds, especially six-membered pyrimidine ring and their derivatives have occupied an important place among organic compounds owing to their various biological signifiances (Zheng *et al.*, 2007). They have been explored for the development of various pharmaceutically important compounds because of their medicinal values (Jain *et al.*, 2006). They also form the basic structural fragment of many alkaloids and pharmacologically active compounds. As part of our ongoing programme on bioactive pyrimidine molecules, we have synthesized a new pyrimidine compound starting from an important building block namely 6-Amino- 1,3-dimethylpyrimidine-2,4(1*H*, 3H)-dione (Blicke & Godt, 1954; Das *et al.*, 2008). The title  compound  crystallizes with two molecules in the asymmetric unit. The two uracil moieties in the same molecule are connected by a pair of strong N—H···O hydrogen bonds. The packing   is stabilized by N—H···.O, C—H···.O and C—H···.N interactions.

### Experimental

Distilled water was added in excess to 6-Amino-1,3-dimethylpyrimidine-2,4(1*H*,3H) -dione (2 mmol) in order to dissolve it  completely in a stirring condition. Then, *p*-chlorobenzaldehyde was added until a precipitate appeared. After 1 hr of stirring the product was filtered and purified by column chromatography. The product was recrystallized by using distilled ethanol, yielding suitable crystals for data collection.

### Refinement

All   hydrogen atoms   were   found in  difference Fourier maps. They were refined using a riding model with N-H = 0.86 Å, C_aromatic_-H = 0.93 Å, C_methyl_-H = 0.96 Å, C_tertiary_-H = 0.98 Å and with U(H) set to 1.2 U_eq_(C,N) or 1.5 U_eq_(C_methyl_).

### Crystal data

**Table:**

C_19_H_21_ClN_6_O_4_	*D*_x_ = 1.472 Mg m^−^^3^
*M**_r_* = 432.87	Mo *K*α radiation, λ = 0.71073 Å
Orthorhombic, *P*2_1_2_1_2_1_	Cell parameters from 9985 reflections
*a* = 11.5365 (3) Å	θ = 2.2–25.7°
*b* = 14.5935 (4) Å	µ = 0.24 mm^−^^1^
*c* = 23.2102 (6) Å	*T* = 293 K
*V* = 3907.62 (18) Å^3^	Blocks, colorless
*Z* = 8	0.40 × 0.28 × 0.16 mm
*F*(000) = 1808	

### Data collection

**Table:**

Bruker SMART CCD area-detector diffractometer	6901 reflections with *I* > 2σ(*I*)
Radiation source: fine-focus sealed tube	*R*_int_ = 0.038
graphite	θ_max_ = 28.4°, θ_min_ = 1.7°
φ and ω scans	*h* = −15→15
53787 measured reflections	*k* = −19→19
9742 independent reflections	*l* = −30→30

### Refinement

**Table:**

Refinement on *F*^2^	Secondary atom site location: difference Fourier map
Least-squares matrix: full	Hydrogen site location: inferred from neighbouring sites
*R*[*F*^2^ > 2σ(*F*^2^)] = 0.056	H-atom parameters constrained
*wR*(*F*^2^) = 0.166	*w* = 1/[σ^2^(*F*_o_^2^) + (0.09*P*)^2^ + 0.7964*P*] where *P* = (*F*_o_^2^ + 2*F*_c_^2^)/3
*S* = 1.06	(Δ/σ)_max_ < 0.001
9742 reflections	Δρ_max_ = 1.27 e Å^−^^3^
549 parameters	Δρ_min_ = −0.28 e Å^−^^3^
0 restraints	Absolute structure: Flack (1983), 4345 Friedel pairs
Primary atom site location: structure-invariant direct methods	Flack parameter: 0.15 (8)

### Special details

**Table:**

Geometry. All s.u.'s (except the s.u. in the dihedral angle between two l.s. planes) are estimated using the full covariance matrix. The cell s.u.'s are taken into account individually in the estimation of s.u.'s in distances, angles and torsion angles; correlations between s.u.'s in cell parameters are only used when they are defined by crystal symmetry. An approximate (isotropic) treatment of cell s.u.'s is used for estimating s.u.'s involving l.s. planes.
Refinement. Refinement of *F*^2^ against ALL reflections. The weighted *R*-factor *wR* and goodness of fit *S* are based on *F*^2^, conventional *R*-factors *R* are based on *F*, with *F* set to zero for negative *F*^2^. The threshold expression of *F*^2^ > 2σ(*F*^2^) is used only for calculating *R*-factors(gt) *etc*. and is not relevant to the choice of reflections for refinement. *R*-factors based on *F*^2^ are statistically about twice as large as those based on *F*, and *R*- factors based on ALL data will be even larger.

### Fractional atomic coordinates and isotropic or equivalent isotropic displacement parameters (Å^2^)

**Table:**

	*x*	*y*	*z*	*U*_iso_*/*U*_eq_	
Cl1	0.87268 (9)	0.84719 (7)	0.14783 (4)	0.0691 (3)	
Cl2	0.62621 (10)	0.67886 (8)	0.36362 (6)	0.0842 (4)	
O8	1.06232 (19)	0.88990 (14)	0.27567 (9)	0.0449 (5)	
N10	0.9975 (2)	1.12225 (16)	0.18849 (10)	0.0409 (6)	
C27	0.9774 (3)	1.0599 (2)	0.38507 (12)	0.0388 (6)	
O1	0.43437 (19)	0.63236 (14)	0.23040 (9)	0.0462 (5)	
N11	1.0865 (2)	0.97903 (16)	0.19627 (10)	0.0384 (5)	
N6	0.3816 (2)	0.59997 (17)	0.11616 (11)	0.0452 (6)	
H6A	0.3264	0.6301	0.1000	0.054*	
H6B	0.4096	0.6184	0.1485	0.054*	
N2	0.4159 (2)	0.53819 (17)	0.30849 (10)	0.0392 (6)	
C11	0.4596 (3)	0.55679 (19)	0.25331 (12)	0.0362 (6)	
C14	0.5145 (3)	0.4746 (2)	0.11458 (12)	0.0403 (7)	
C36	1.0386 (2)	0.96383 (19)	0.25117 (12)	0.0351 (6)	
C3	0.6561 (3)	0.6419 (2)	0.11131 (12)	0.0402 (6)	
H3	0.6126	0.6229	0.0798	0.048*	
N1	0.5063 (2)	0.39511 (16)	0.31134 (10)	0.0386 (6)	
C10	0.4440 (3)	0.4624 (2)	0.34031 (13)	0.0396 (6)	
O7	1.0992 (2)	1.06161 (18)	0.11334 (10)	0.0649 (7)	
O6	0.8555 (2)	1.19062 (15)	0.38902 (10)	0.0514 (5)	
C9	0.5423 (2)	0.40516 (19)	0.25483 (11)	0.0338 (6)	
C1	0.5849 (3)	0.50156 (19)	0.16763 (12)	0.0371 (6)	
H1	0.6471	0.4558	0.1680	0.044*	
C30	0.9395 (3)	1.1447 (2)	0.40763 (13)	0.0421 (7)	
N9	1.1133 (2)	0.93617 (19)	0.38994 (12)	0.0477 (6)	
H9A	1.1693	0.9094	0.4076	0.057*	
H9B	1.0852	0.9126	0.3590	0.057*	
C20	0.9093 (2)	1.02501 (19)	0.33366 (12)	0.0353 (6)	
H20	0.8455	1.0691	0.3310	0.042*	
C34	0.9553 (3)	1.11502 (19)	0.24430 (12)	0.0355 (6)	
O4	0.6321 (3)	0.34234 (19)	0.10500 (11)	0.0808 (10)	
C33	0.9683 (3)	1.03421 (19)	0.27454 (12)	0.0345 (6)	
N3	0.5937 (2)	0.33165 (16)	0.23116 (11)	0.0435 (6)	
H3A	0.6195	0.3343	0.1964	0.052*	
H3B	0.6009	0.2819	0.2507	0.052*	
N5	0.3759 (2)	0.49787 (19)	0.03907 (11)	0.0450 (6)	
O2	0.4133 (3)	0.45193 (18)	0.38953 (10)	0.0665 (7)	
C17	0.4243 (3)	0.5239 (2)	0.09087 (13)	0.0398 (7)	
N12	0.9021 (2)	1.18920 (17)	0.26519 (11)	0.0446 (6)	
H12A	0.8735	1.1884	0.2994	0.054*	
H12B	0.8965	1.2378	0.2444	0.054*	
C8	0.5272 (3)	0.48760 (19)	0.22656 (12)	0.0357 (6)	
C24	0.7074 (3)	0.7778 (2)	0.35625 (15)	0.0497 (8)	
C21	0.8455 (2)	0.93356 (19)	0.34220 (12)	0.0347 (6)	
N8	0.9983 (2)	1.1785 (2)	0.45592 (11)	0.0516 (7)	
C2	0.6515 (2)	0.59179 (19)	0.16235 (12)	0.0369 (6)	
N7	1.1188 (2)	1.0487 (2)	0.46059 (11)	0.0511 (7)	
C35	1.0637 (3)	1.0546 (2)	0.16289 (13)	0.0437 (7)	
C4	0.7240 (3)	0.7190 (2)	0.10674 (14)	0.0450 (7)	
H4	0.7253	0.7519	0.0724	0.054*	
O5	1.1412 (2)	1.1681 (2)	0.52252 (11)	0.0755 (8)	
C7	0.7218 (3)	0.6211 (2)	0.20748 (13)	0.0447 (7)	
H7	0.7219	0.5882	0.2418	0.054*	
C22	0.7752 (3)	0.9018 (2)	0.29750 (13)	0.0407 (7)	
H22	0.7749	0.9329	0.2625	0.049*	
C26	0.8422 (3)	0.8873 (2)	0.39395 (13)	0.0405 (7)	
H26	0.8865	0.9085	0.4247	0.049*	
C12	0.5385 (3)	0.3124 (2)	0.34431 (14)	0.0515 (8)	
H12C	0.4977	0.2604	0.3291	0.077*	
H12D	0.5181	0.3207	0.3841	0.077*	
H12E	0.6204	0.3023	0.3412	0.077*	
O3	0.3531 (3)	0.3928 (2)	−0.03150 (11)	0.0852 (9)	
C23	0.7055 (3)	0.8249 (2)	0.30412 (14)	0.0471 (7)	
H23	0.6583	0.8052	0.2741	0.057*	
C5	0.7897 (3)	0.7477 (2)	0.15242 (14)	0.0436 (7)	
C15	0.5501 (4)	0.3921 (3)	0.08698 (13)	0.0562 (9)	
C6	0.7909 (3)	0.6971 (2)	0.20284 (14)	0.0488 (8)	
H6	0.8382	0.7146	0.2334	0.059*	
C16	0.4053 (4)	0.4160 (3)	0.01292 (15)	0.0588 (10)	
C25	0.7737 (3)	0.8091 (2)	0.40128 (15)	0.0487 (8)	
H25	0.7731	0.7785	0.4364	0.058*	
N4	0.4919 (3)	0.3658 (2)	0.03778 (11)	0.0617 (9)	
C29	1.0896 (3)	1.1343 (3)	0.48273 (14)	0.0561 (9)	
C28	1.0693 (3)	1.0151 (2)	0.41075 (13)	0.0432 (7)	
C37	0.9705 (4)	1.2031 (2)	0.15333 (14)	0.0581 (9)	
H37A	1.0169	1.2539	0.1659	0.087*	
H37B	0.8899	1.2181	0.1575	0.087*	
H37C	0.9870	1.1901	0.1136	0.087*	
C13	0.3377 (3)	0.6053 (2)	0.33519 (14)	0.0520 (8)	
H13A	0.2920	0.5757	0.3643	0.078*	
H13B	0.2875	0.6307	0.3063	0.078*	
H13C	0.3825	0.6535	0.3524	0.078*	
C38	1.1673 (3)	0.9112 (2)	0.17263 (15)	0.0513 (8)	
H38A	1.1296	0.8771	0.1426	0.077*	
H38B	1.1914	0.8702	0.2027	0.077*	
H38C	1.2339	0.9420	0.1571	0.077*	
C19	0.2869 (3)	0.5538 (3)	0.00979 (15)	0.0596 (9)	
H19A	0.2140	0.5468	0.0292	0.089*	
H19B	0.2792	0.5340	−0.0295	0.089*	
H19C	0.3096	0.6171	0.0106	0.089*	
C31	1.2062 (4)	0.9963 (3)	0.49141 (17)	0.0708 (12)	
H31A	1.1736	0.9392	0.5039	0.106*	
H31B	1.2320	1.0305	0.5243	0.106*	
H31C	1.2708	0.9847	0.4664	0.106*	
C32	0.9631 (4)	1.2684 (3)	0.47975 (17)	0.0706 (12)	
H32A	0.9899	1.2736	0.5188	0.106*	
H32B	0.8801	1.2733	0.4790	0.106*	
H32C	0.9963	1.3166	0.4569	0.106*	
C18	0.5258 (6)	0.2797 (3)	0.01056 (19)	0.109 (2)	
H18A	0.4577	0.2460	−0.0002	0.163*	
H18B	0.5710	0.2441	0.0371	0.163*	
H18C	0.5712	0.2924	−0.0232	0.163*	

### Atomic displacement parameters (Å^2^)

**Table:**

	*U*^11^	*U*^22^	*U*^33^	*U*^12^	*U*^13^	*U*^23^
Cl1	0.0614 (6)	0.0759 (6)	0.0700 (6)	−0.0308 (5)	0.0047 (5)	−0.0041 (5)
Cl2	0.0732 (7)	0.0615 (6)	0.1179 (9)	−0.0344 (5)	−0.0091 (6)	0.0144 (6)
O8	0.0568 (14)	0.0364 (11)	0.0415 (11)	0.0065 (10)	0.0041 (10)	−0.0002 (9)
N10	0.0547 (15)	0.0348 (12)	0.0331 (12)	−0.0055 (11)	0.0044 (11)	0.0006 (10)
C27	0.0420 (16)	0.0391 (15)	0.0353 (14)	−0.0089 (13)	0.0064 (13)	−0.0026 (12)
O1	0.0599 (14)	0.0363 (11)	0.0422 (11)	0.0113 (10)	0.0016 (10)	0.0011 (9)
N11	0.0389 (13)	0.0387 (13)	0.0374 (13)	−0.0029 (10)	0.0045 (11)	−0.0033 (10)
N6	0.0435 (14)	0.0437 (14)	0.0484 (14)	0.0042 (11)	−0.0080 (12)	−0.0082 (12)
N2	0.0410 (13)	0.0387 (13)	0.0380 (13)	0.0004 (11)	0.0074 (11)	−0.0024 (10)
C11	0.0424 (16)	0.0326 (14)	0.0334 (14)	−0.0011 (12)	0.0020 (13)	0.0027 (11)
C14	0.0546 (18)	0.0343 (14)	0.0321 (14)	0.0010 (13)	0.0075 (13)	−0.0004 (11)
C36	0.0352 (15)	0.0343 (14)	0.0357 (14)	−0.0028 (12)	−0.0006 (12)	−0.0055 (11)
C3	0.0405 (16)	0.0462 (16)	0.0340 (14)	−0.0016 (13)	−0.0024 (13)	0.0045 (13)
N1	0.0491 (15)	0.0311 (12)	0.0357 (12)	−0.0046 (10)	0.0015 (11)	0.0034 (10)
C10	0.0431 (16)	0.0417 (16)	0.0339 (15)	−0.0028 (13)	0.0063 (13)	0.0013 (12)
O7	0.0772 (17)	0.0715 (17)	0.0459 (13)	0.0076 (14)	0.0232 (13)	0.0079 (12)
O6	0.0567 (14)	0.0451 (12)	0.0525 (13)	−0.0015 (11)	0.0085 (11)	−0.0105 (10)
C9	0.0357 (15)	0.0337 (14)	0.0320 (14)	−0.0027 (11)	−0.0005 (12)	0.0007 (11)
C1	0.0434 (16)	0.0329 (14)	0.0349 (14)	0.0083 (12)	0.0024 (12)	0.0035 (11)
C30	0.0435 (17)	0.0425 (16)	0.0404 (16)	−0.0154 (14)	0.0115 (13)	−0.0097 (13)
N9	0.0459 (15)	0.0518 (15)	0.0455 (14)	−0.0006 (12)	−0.0062 (12)	−0.0039 (12)
C20	0.0351 (14)	0.0348 (14)	0.0360 (14)	−0.0038 (11)	0.0029 (12)	−0.0021 (11)
C34	0.0368 (15)	0.0326 (14)	0.0371 (14)	−0.0045 (11)	−0.0003 (12)	−0.0028 (11)
O4	0.137 (3)	0.0604 (16)	0.0452 (13)	0.0466 (18)	−0.0001 (16)	−0.0088 (12)
C33	0.0406 (16)	0.0334 (14)	0.0296 (13)	−0.0047 (12)	0.0039 (12)	−0.0051 (11)
N3	0.0613 (16)	0.0313 (12)	0.0380 (13)	0.0064 (12)	0.0050 (12)	0.0043 (10)
N5	0.0443 (15)	0.0530 (15)	0.0377 (13)	−0.0016 (12)	0.0070 (11)	−0.0035 (12)
O2	0.094 (2)	0.0638 (16)	0.0421 (13)	0.0103 (14)	0.0229 (14)	0.0082 (12)
C17	0.0417 (16)	0.0396 (15)	0.0381 (15)	−0.0061 (13)	0.0102 (13)	0.0025 (12)
N12	0.0573 (16)	0.0337 (12)	0.0430 (13)	0.0029 (12)	0.0063 (12)	0.0026 (11)
C8	0.0404 (16)	0.0322 (14)	0.0347 (14)	0.0013 (12)	0.0014 (12)	0.0024 (11)
C24	0.0365 (16)	0.0418 (16)	0.071 (2)	−0.0085 (13)	0.0025 (16)	0.0015 (16)
C21	0.0331 (14)	0.0334 (14)	0.0375 (14)	−0.0017 (11)	0.0027 (12)	−0.0010 (11)
N8	0.0562 (17)	0.0572 (17)	0.0415 (14)	−0.0141 (14)	0.0102 (13)	−0.0173 (13)
C2	0.0369 (15)	0.0382 (15)	0.0355 (14)	0.0087 (12)	0.0068 (12)	0.0019 (11)
N7	0.0448 (15)	0.0738 (19)	0.0347 (13)	−0.0116 (14)	−0.0010 (12)	−0.0082 (13)
C35	0.0463 (17)	0.0463 (17)	0.0386 (16)	−0.0049 (13)	0.0045 (14)	−0.0058 (13)
C4	0.0391 (16)	0.0514 (18)	0.0446 (16)	−0.0070 (14)	0.0058 (14)	0.0097 (14)
O5	0.0670 (17)	0.109 (2)	0.0505 (14)	−0.0212 (17)	−0.0030 (13)	−0.0290 (15)
C7	0.0473 (18)	0.0517 (18)	0.0351 (15)	0.0068 (15)	−0.0039 (13)	0.0084 (14)
C22	0.0403 (16)	0.0426 (16)	0.0393 (15)	−0.0019 (13)	−0.0062 (13)	−0.0013 (13)
C26	0.0352 (15)	0.0451 (16)	0.0411 (15)	−0.0064 (13)	−0.0025 (13)	0.0019 (13)
C12	0.077 (2)	0.0342 (15)	0.0434 (16)	0.0047 (15)	0.0043 (17)	0.0115 (13)
O3	0.111 (2)	0.097 (2)	0.0483 (15)	0.003 (2)	−0.0025 (16)	−0.0330 (15)
C23	0.0373 (16)	0.0462 (18)	0.0578 (19)	−0.0046 (14)	−0.0075 (14)	−0.0057 (15)
C5	0.0323 (15)	0.0494 (17)	0.0492 (17)	−0.0044 (13)	0.0040 (14)	−0.0009 (14)
C15	0.083 (3)	0.052 (2)	0.0332 (16)	0.0119 (19)	0.0099 (17)	0.0019 (14)
C6	0.0408 (17)	0.058 (2)	0.0476 (18)	0.0013 (15)	−0.0088 (14)	−0.0032 (15)
C16	0.069 (2)	0.069 (2)	0.0384 (17)	−0.009 (2)	0.0109 (17)	−0.0094 (17)
C25	0.0457 (17)	0.0458 (18)	0.0546 (19)	−0.0040 (14)	−0.0005 (15)	0.0139 (15)
N4	0.099 (3)	0.0522 (17)	0.0336 (13)	0.0071 (17)	0.0081 (15)	−0.0124 (13)
C29	0.059 (2)	0.074 (2)	0.0352 (16)	−0.0236 (19)	0.0099 (16)	−0.0163 (17)
C28	0.0427 (17)	0.0527 (19)	0.0341 (15)	−0.0165 (14)	0.0045 (13)	−0.0025 (13)
C37	0.089 (3)	0.0460 (19)	0.0389 (16)	−0.0015 (18)	0.0128 (18)	0.0104 (14)
C13	0.0527 (19)	0.0535 (19)	0.0497 (18)	0.0154 (16)	0.0073 (16)	−0.0052 (15)
C38	0.0471 (18)	0.054 (2)	0.0532 (19)	0.0068 (15)	0.0106 (16)	−0.0085 (15)
C19	0.052 (2)	0.084 (3)	0.0426 (17)	−0.0011 (19)	−0.0005 (16)	0.0002 (18)
C31	0.064 (3)	0.093 (3)	0.055 (2)	−0.001 (2)	−0.0132 (19)	−0.010 (2)
C32	0.084 (3)	0.065 (2)	0.063 (2)	−0.016 (2)	0.021 (2)	−0.035 (2)
C18	0.193 (6)	0.074 (3)	0.059 (3)	0.043 (4)	0.000 (3)	−0.032 (2)

### Geometric parameters (Å, °)

**Table:**

Cl1—C5	1.743 (3)	N12—H12A	0.8600
Cl2—C24	1.730 (3)	N12—H12B	0.8600
O8—C36	1.250 (3)	C24—C25	1.373 (5)
N10—C35	1.383 (4)	C24—C23	1.392 (5)
N10—C34	1.388 (4)	C21—C26	1.379 (4)
N10—C37	1.468 (4)	C21—C22	1.396 (4)
C27—C28	1.381 (5)	N8—C29	1.383 (5)
C27—C30	1.414 (4)	N8—C32	1.481 (4)
C27—C20	1.517 (4)	C2—C7	1.392 (4)
O1—C11	1.258 (3)	N7—C28	1.380 (4)
N11—C35	1.373 (4)	N7—C29	1.393 (5)
N11—C36	1.406 (4)	N7—C31	1.454 (5)
N11—C38	1.466 (4)	C4—C5	1.369 (4)
N6—C17	1.349 (4)	C4—H4	0.9300
N6—H6A	0.8600	O5—C29	1.204 (4)
N6—H6B	0.8600	C7—C6	1.369 (5)
N2—C10	1.369 (4)	C7—H7	0.9300
N2—C11	1.403 (4)	C22—C23	1.388 (4)
N2—C13	1.469 (4)	C22—H22	0.9300
C11—C8	1.419 (4)	C26—C25	1.397 (4)
C14—C17	1.380 (4)	C26—H26	0.9300
C14—C15	1.424 (4)	C12—H12C	0.9600
C14—C1	1.527 (4)	C12—H12D	0.9600
C36—C33	1.417 (4)	C12—H12E	0.9600
C3—C4	1.376 (4)	O3—C16	1.241 (4)
C3—C2	1.393 (4)	C23—H23	0.9300
C3—H3	0.9300	C5—C6	1.383 (4)
N1—C9	1.384 (4)	C15—N4	1.379 (5)
N1—C10	1.391 (4)	C6—H6	0.9300
N1—C12	1.477 (4)	C16—N4	1.367 (5)
C10—O2	1.206 (4)	C25—H25	0.9300
O7—C35	1.225 (4)	N4—C18	1.460 (5)
O6—C30	1.254 (4)	C37—H37A	0.9600
C9—N3	1.343 (4)	C37—H37B	0.9600
C9—C8	1.381 (4)	C37—H37C	0.9600
C1—C2	1.530 (4)	C13—H13A	0.9600
C1—C8	1.535 (4)	C13—H13B	0.9600
C1—H1	0.9800	C13—H13C	0.9600
C30—N8	1.400 (4)	C38—H38A	0.9600
N9—C28	1.347 (4)	C38—H38B	0.9600
N9—H9A	0.8600	C38—H38C	0.9600
N9—H9B	0.8600	C19—H19A	0.9600
C20—C21	1.537 (4)	C19—H19B	0.9600
C20—C33	1.538 (4)	C19—H19C	0.9600
C20—H20	0.9800	C31—H31A	0.9600
C34—N12	1.335 (4)	C31—H31B	0.9600
C34—C33	1.380 (4)	C31—H31C	0.9600
O4—C15	1.264 (5)	C32—H32A	0.9600
N3—H3A	0.8600	C32—H32B	0.9600
N3—H3B	0.8600	C32—H32C	0.9600
N5—C17	1.379 (4)	C18—H18A	0.9600
N5—C16	1.382 (5)	C18—H18B	0.9600
N5—C19	1.478 (5)	C18—H18C	0.9600
			
C35—N10—C34	122.7 (2)	O7—C35—N11	122.2 (3)
C35—N10—C37	116.9 (2)	O7—C35—N10	121.9 (3)
C34—N10—C37	120.4 (3)	N11—C35—N10	115.9 (2)
C28—C27—C30	119.5 (3)	C5—C4—C3	120.4 (3)
C28—C27—C20	125.3 (3)	C5—C4—H4	119.8
C30—C27—C20	115.1 (3)	C3—C4—H4	119.8
C35—N11—C36	124.2 (2)	C6—C7—C2	121.9 (3)
C35—N11—C38	116.9 (3)	C6—C7—H7	119.0
C36—N11—C38	118.8 (3)	C2—C7—H7	119.0
C17—N6—H6A	120.0	C23—C22—C21	121.5 (3)
C17—N6—H6B	120.0	C23—C22—H22	119.3
H6A—N6—H6B	120.0	C21—C22—H22	119.3
C10—N2—C11	124.4 (2)	C21—C26—C25	121.4 (3)
C10—N2—C13	117.2 (2)	C21—C26—H26	119.3
C11—N2—C13	118.5 (2)	C25—C26—H26	119.3
O1—C11—N2	118.2 (3)	N1—C12—H12C	109.5
O1—C11—C8	124.5 (3)	N1—C12—H12D	109.5
N2—C11—C8	117.3 (2)	H12C—C12—H12D	109.5
C17—C14—C15	118.6 (3)	N1—C12—H12E	109.5
C17—C14—C1	126.1 (3)	H12C—C12—H12E	109.5
C15—C14—C1	115.3 (3)	H12D—C12—H12E	109.5
O8—C36—N11	117.5 (3)	C22—C23—C24	119.1 (3)
O8—C36—C33	125.2 (3)	C22—C23—H23	120.5
N11—C36—C33	117.2 (3)	C24—C23—H23	120.5
C4—C3—C2	121.1 (3)	C4—C5—C6	119.9 (3)
C4—C3—H3	119.4	C4—C5—Cl1	120.8 (2)
C2—C3—H3	119.4	C6—C5—Cl1	119.4 (2)
C9—N1—C10	122.6 (2)	O4—C15—N4	118.6 (3)
C9—N1—C12	120.2 (3)	O4—C15—C14	123.6 (3)
C10—N1—C12	117.2 (2)	N4—C15—C14	117.9 (3)
O2—C10—N2	123.0 (3)	C7—C6—C5	119.5 (3)
O2—C10—N1	121.4 (3)	C7—C6—H6	120.2
N2—C10—N1	115.6 (2)	C5—C6—H6	120.2
N3—C9—C8	123.8 (3)	O3—C16—N4	124.0 (4)
N3—C9—N1	115.8 (2)	O3—C16—N5	118.8 (4)
C8—C9—N1	120.3 (3)	N4—C16—N5	117.2 (3)
C14—C1—C2	115.1 (2)	C24—C25—C26	119.6 (3)
C14—C1—C8	117.0 (2)	C24—C25—H25	120.2
C2—C1—C8	113.8 (2)	C26—C25—H25	120.2
C14—C1—H1	102.7	C16—N4—C15	123.8 (3)
C2—C1—H1	102.7	C16—N4—C18	118.4 (4)
C8—C1—H1	102.7	C15—N4—C18	117.9 (4)
O6—C30—N8	117.5 (3)	O5—C29—N8	122.1 (4)
O6—C30—C27	125.4 (3)	O5—C29—N7	122.1 (4)
N8—C30—C27	117.0 (3)	N8—C29—N7	115.9 (3)
C28—N9—H9A	120.0	N9—C28—N7	116.7 (3)
C28—N9—H9B	120.0	N9—C28—C27	122.6 (3)
H9A—N9—H9B	120.0	N7—C28—C27	120.7 (3)
C27—C20—C21	116.0 (2)	N10—C37—H37A	109.5
C27—C20—C33	116.3 (2)	N10—C37—H37B	109.5
C21—C20—C33	113.8 (2)	H37A—C37—H37B	109.5
C27—C20—H20	102.6	N10—C37—H37C	109.5
C21—C20—H20	102.6	H37A—C37—H37C	109.5
C33—C20—H20	102.6	H37B—C37—H37C	109.5
N12—C34—C33	123.9 (3)	N2—C13—H13A	109.5
N12—C34—N10	116.0 (2)	N2—C13—H13B	109.5
C33—C34—N10	120.1 (3)	H13A—C13—H13B	109.5
C34—C33—C36	119.1 (3)	N2—C13—H13C	109.5
C34—C33—C20	118.7 (3)	H13A—C13—H13C	109.5
C36—C33—C20	122.1 (3)	H13B—C13—H13C	109.5
C9—N3—H3A	120.0	N11—C38—H38A	109.5
C9—N3—H3B	120.0	N11—C38—H38B	109.5
H3A—N3—H3B	120.0	H38A—C38—H38B	109.5
C17—N5—C16	121.5 (3)	N11—C38—H38C	109.5
C17—N5—C19	122.0 (3)	H38A—C38—H38C	109.5
C16—N5—C19	116.5 (3)	H38B—C38—H38C	109.5
N6—C17—N5	117.3 (3)	N5—C19—H19A	109.5
N6—C17—C14	122.1 (3)	N5—C19—H19B	109.5
N5—C17—C14	120.6 (3)	H19A—C19—H19B	109.5
C34—N12—H12A	120.0	N5—C19—H19C	109.5
C34—N12—H12B	120.0	H19A—C19—H19C	109.5
H12A—N12—H12B	120.0	H19B—C19—H19C	109.5
C9—C8—C11	118.8 (3)	N7—C31—H31A	109.5
C9—C8—C1	119.0 (3)	N7—C31—H31B	109.5
C11—C8—C1	122.3 (2)	H31A—C31—H31B	109.5
C25—C24—C23	120.4 (3)	N7—C31—H31C	109.5
C25—C24—Cl2	120.3 (3)	H31A—C31—H31C	109.5
C23—C24—Cl2	119.3 (3)	H31B—C31—H31C	109.5
C26—C21—C22	117.9 (3)	N8—C32—H32A	109.5
C26—C21—C20	123.4 (3)	N8—C32—H32B	109.5
C22—C21—C20	118.1 (3)	H32A—C32—H32B	109.5
C29—N8—C30	124.4 (3)	N8—C32—H32C	109.5
C29—N8—C32	117.0 (3)	H32A—C32—H32C	109.5
C30—N8—C32	118.5 (3)	H32B—C32—H32C	109.5
C7—C2—C3	117.1 (3)	N4—C18—H18A	109.5
C7—C2—C1	119.8 (3)	N4—C18—H18B	109.5
C3—C2—C1	122.6 (3)	H18A—C18—H18B	109.5
C28—N7—C29	121.9 (3)	N4—C18—H18C	109.5
C28—N7—C31	120.8 (3)	H18A—C18—H18C	109.5
C29—N7—C31	117.2 (3)	H18B—C18—H18C	109.5
			
C10—N2—C11—O1	−174.5 (3)	C27—C20—C21—C22	175.6 (3)
C13—N2—C11—O1	3.5 (4)	C33—C20—C21—C22	−45.5 (4)
C10—N2—C11—C8	7.0 (4)	O6—C30—N8—C29	177.5 (3)
C13—N2—C11—C8	−175.0 (3)	C27—C30—N8—C29	−0.4 (4)
C35—N11—C36—O8	176.5 (3)	O6—C30—N8—C32	−3.7 (4)
C38—N11—C36—O8	−4.3 (4)	C27—C30—N8—C32	178.3 (3)
C35—N11—C36—C33	−4.1 (4)	C4—C3—C2—C7	−2.5 (4)
C38—N11—C36—C33	175.0 (3)	C4—C3—C2—C1	−174.9 (3)
C11—N2—C10—O2	173.3 (3)	C14—C1—C2—C7	−177.7 (3)
C13—N2—C10—O2	−4.7 (5)	C8—C1—C2—C7	43.3 (4)
C11—N2—C10—N1	−9.5 (4)	C14—C1—C2—C3	−5.4 (4)
C13—N2—C10—N1	172.4 (3)	C8—C1—C2—C3	−144.4 (3)
C9—N1—C10—O2	179.8 (3)	C36—N11—C35—O7	−173.6 (3)
C12—N1—C10—O2	−3.0 (5)	C38—N11—C35—O7	7.2 (5)
C9—N1—C10—N2	2.6 (4)	C36—N11—C35—N10	6.0 (4)
C12—N1—C10—N2	179.8 (3)	C38—N11—C35—N10	−173.2 (3)
C10—N1—C9—N3	−175.0 (3)	C34—N10—C35—O7	179.2 (3)
C12—N1—C9—N3	7.9 (4)	C37—N10—C35—O7	0.5 (5)
C10—N1—C9—C8	6.6 (4)	C34—N10—C35—N11	−0.5 (4)
C12—N1—C9—C8	−170.5 (3)	C37—N10—C35—N11	−179.1 (3)
C17—C14—C1—C2	−61.2 (4)	C2—C3—C4—C5	0.7 (5)
C15—C14—C1—C2	116.5 (3)	C3—C2—C7—C6	1.5 (4)
C17—C14—C1—C8	76.4 (4)	C1—C2—C7—C6	174.2 (3)
C15—C14—C1—C8	−105.8 (3)	C26—C21—C22—C23	−1.3 (4)
C28—C27—C30—O6	−178.1 (3)	C20—C21—C22—C23	−173.2 (3)
C20—C27—C30—O6	0.8 (4)	C22—C21—C26—C25	2.1 (4)
C28—C27—C30—N8	−0.3 (4)	C20—C21—C26—C25	173.5 (3)
C20—C27—C30—N8	178.6 (2)	C21—C22—C23—C24	−1.0 (5)
C28—C27—C20—C21	61.2 (4)	C25—C24—C23—C22	2.6 (5)
C30—C27—C20—C21	−117.7 (3)	Cl2—C24—C23—C22	−176.9 (2)
C28—C27—C20—C33	−76.6 (4)	C3—C4—C5—C6	2.2 (5)
C30—C27—C20—C33	104.5 (3)	C3—C4—C5—Cl1	−178.1 (2)
C35—N10—C34—N12	173.9 (3)	C17—C14—C15—O4	179.5 (3)
C37—N10—C34—N12	−7.6 (4)	C1—C14—C15—O4	1.6 (5)
C35—N10—C34—C33	−6.8 (4)	C17—C14—C15—N4	−0.1 (5)
C37—N10—C34—C33	171.7 (3)	C1—C14—C15—N4	−178.1 (3)
N12—C34—C33—C36	−172.1 (3)	C2—C7—C6—C5	1.3 (5)
N10—C34—C33—C36	8.6 (4)	C4—C5—C6—C7	−3.1 (5)
N12—C34—C33—C20	5.7 (4)	Cl1—C5—C6—C7	177.1 (2)
N10—C34—C33—C20	−173.5 (2)	C17—N5—C16—O3	174.9 (3)
O8—C36—C33—C34	175.9 (3)	C19—N5—C16—O3	−2.8 (5)
N11—C36—C33—C34	−3.4 (4)	C17—N5—C16—N4	−5.8 (5)
O8—C36—C33—C20	−1.9 (4)	C19—N5—C16—N4	176.6 (3)
N11—C36—C33—C20	178.8 (2)	C23—C24—C25—C26	−1.9 (5)
C27—C20—C33—C34	−86.8 (3)	Cl2—C24—C25—C26	177.6 (2)
C21—C20—C33—C34	134.4 (3)	C21—C26—C25—C24	−0.5 (5)
C27—C20—C33—C36	90.9 (3)	O3—C16—N4—C15	179.6 (4)
C21—C20—C33—C36	−47.8 (4)	N5—C16—N4—C15	0.3 (5)
C16—N5—C17—N6	−173.1 (3)	O3—C16—N4—C18	0.2 (6)
C19—N5—C17—N6	4.4 (4)	N5—C16—N4—C18	−179.1 (4)
C16—N5—C17—C14	8.3 (4)	O4—C15—N4—C16	−177.1 (4)
C19—N5—C17—C14	−174.1 (3)	C14—C15—N4—C16	2.6 (5)
C15—C14—C17—N6	176.4 (3)	O4—C15—N4—C18	2.3 (6)
C1—C14—C17—N6	−5.9 (5)	C14—C15—N4—C18	−178.0 (4)
C15—C14—C17—N5	−5.2 (4)	C30—N8—C29—O5	176.4 (3)
C1—C14—C17—N5	172.5 (3)	C32—N8—C29—O5	−2.4 (5)
N3—C9—C8—C11	172.6 (3)	C30—N8—C29—N7	−3.1 (4)
N1—C9—C8—C11	−9.1 (4)	C32—N8—C29—N7	178.1 (3)
N3—C9—C8—C1	−7.9 (5)	C28—N7—C29—O5	−171.8 (3)
N1—C9—C8—C1	170.4 (3)	C31—N7—C29—O5	6.6 (5)
O1—C11—C8—C9	−175.7 (3)	C28—N7—C29—N8	7.7 (4)
N2—C11—C8—C9	2.7 (4)	C31—N7—C29—N8	−173.8 (3)
O1—C11—C8—C1	4.7 (5)	C29—N7—C28—N9	172.9 (3)
N2—C11—C8—C1	−176.8 (3)	C31—N7—C28—N9	−5.6 (4)
C14—C1—C8—C9	90.4 (3)	C29—N7—C28—C27	−8.8 (4)
C2—C1—C8—C9	−131.4 (3)	C31—N7—C28—C27	172.8 (3)
C14—C1—C8—C11	−90.1 (3)	C30—C27—C28—N9	−176.9 (3)
C2—C1—C8—C11	48.1 (4)	C20—C27—C28—N9	4.2 (5)
C27—C20—C21—C26	4.2 (4)	C30—C27—C28—N7	4.8 (4)
C33—C20—C21—C26	143.1 (3)	C20—C27—C28—N7	−174.0 (3)

### Hydrogen-bond geometry (Å, °)

**Table:**

*D*—H···*A*	*D*—H	H···*A*	*D*···*A*	*D*—H···*A*
N3—H3A···O4	0.86	2.13	2.966 (4)	164
N3—H3B···O1^i^	0.86	2.26	3.059 (3)	154
N6—H6A···O6^i^	0.86	2.29	3.041 (3)	146
N6—H6B···O1	0.86	1.93	2.761 (3)	161
N9—H9A···O4^ii^	0.86	2.51	3.243 (4)	144
N9—H9B···O8	0.86	1.98	2.799 (3)	159
N12—H12A···O6	0.86	2.09	2.924 (3)	163
N12—H12B···O8^ii^	0.86	2.32	3.106 (3)	153
C1—H1···O4	0.98	2.22	2.794 (4)	116
C1—H1···N3	0.98	2.41	2.887 (4)	109
C12—H12C···O1^i^	0.96	2.45	3.164 (4)	131
C12—H12D···O2	0.96	2.27	2.708 (4)	107
C12—H12D···O5^iii^	0.96	2.60	3.322 (4)	133
C13—H13A···O2	0.96	2.36	2.713 (4)	101
C18—H18B···O4	0.96	2.24	2.673 (6)	106
C19—H19B···O3	0.96	2.23	2.650 (5)	105
C20—H20···O6	0.98	2.23	2.807 (4)	116
C20—H20···N12	0.98	2.41	2.876 (4)	108
C31—H31B···O5	0.96	2.27	2.715 (5)	108
C32—H32A···O5	0.96	2.33	2.711 (5)	103
C37—H37A···O8^ii^	0.96	2.57	3.208 (4)	124
C37—H37C···O7	0.96	2.28	2.707 (4)	106
C38—H38B···O8	0.96	2.27	2.699 (4)	106

Symmetry codes: (i) −*x*+1, *y*−1/2, −*z*+1/2; (ii) −*x*+2, *y*+1/2, −*z*+1/2; (iii) *x*−1/2, −*y*+3/2, −*z*+1.
